# Valved Holding Chambers in Young Children With Acute Wheezing: A Randomized Clinical Trial

**DOI:** 10.1001/jamapediatrics.2025.6479

**Published:** 2026-02-23

**Authors:** Péter Csonka, Terhi Ruuska-Loewald, Inka Hämynen, Minna Honkila, Iida Ojaniemi, Eeva Mykkänen, Balázs Kelemen, Minna Juntunen, Salla Kuusela, Marjo Renko, Lauri Lehtimäki, Tytti Pokka, Sauli Palmu

**Affiliations:** 1 Tampere Center for Child, Adolescent and Maternal Health Research, Faculty of Medicine and Health Technology, Tampere University, Tampere, Finland; 2 Terveystalo Healthcare, Finland; 3 Department of Pediatrics and Adolescent Medicine, Oulu University Hospital, Research Unit of Clinical Medicine and Biocenter, University of Oulu, Finland; 4 Kuopio University Hospital, Finland; 5 Oulu University Hospital and Research Unit of Clinical Medicine, University of Oulu, Finland; 6 Department of Pediatrics, Tampere University Hospital, Wellbeing Services of Pirkanmaa, Tampere, Finland; 7 Allergy Centre, Tampere University Hospital, Tampere, Finland; Faculty of Medicine and Health Technology, Tampere University, Tampere, Finland

## Abstract

**Importance:**

Acute wheezing is a leading cause of pediatric emergency visits. Commercially available valved holding chambers (VHCs), used in inhalation therapy, differ in in vitro drug delivery, but the clinical relevance of these differences in young children is uncertain.

**Objective:**

To determine whether a higher-delivery VHC improves clinical outcomes of salbutamol treatment compared with a lower-delivery VHC in young children with acute wheezing.

**Design, Setting, and Participants:**

This randomized clinical trial took place at 4 pediatric emergency departments in Finland with enrollment April 17, 2019, through May 23, 2025 (recruitment pause March 2020 through July 2022), and used intention-to-treat analysis. Patients included children aged 6 to 48 months with moderate or severe wheezing respiratory distress (respiratory distress assessment instrument [RDAI] score of ≥6) who met prespecified inclusion/exclusion criteria. These data were analyzed from May 28, 2025, through September 8, 2025.

**Interventions:**

Salbutamol (dose 0.6 mg per treatment cycle, up to 3-4 cycles at 20-minute intervals) delivered via 1 of 2 commercially available VHCs: either VHC-1 (high in vitro drug delivery) or VHC-2 (low in vitro drug delivery), each used with its respective mask.

**Main Outcomes and Measures:**

Primary outcomes were posttreatment RDAI score and change from baseline. Secondary outcomes included hospitalization rate, the proportion of children requiring a fourth salbutamol dose, and change in respiratory rate and oxygen saturation.

**Results:**

A total of 80 children were randomized and analyzed (mean age, 23.1 months; 36% female and 64% male). Children treated with salbutamol had posttreatment mean (SD) RDAI scores significantly lower in the VHC-1 group (2.7 [2.1]) than in the VHC-2 group (6.8 [3.6]) with a mean difference of −4.1 (95% CI, −5.4 to −2.7; *P* < .001). The mean reduction in RDAI score was greater with VHC-1 than with VHC-2 (mean difference, −5.4; 95% CI, −6.9 to −3.9; *P* < .001). The proportion of children requiring hospitalization was lower in children treated with VHC-1 (20%) than with VHC-2 (50%) (difference, 30%; 95% CI, 9%-49% and number needed to treat, 3.3; 95% CI, 2.1-11; *P* = .005). Relative risk of requiring a fourth salbutamol dose was 0.72 (95% CI, 0.51-0.97; *P* = .03) for VHC-1 vs VHC-2. After treatment, respiratory rate was lower (42 vs 47 breaths per minute; mean difference, −5; 95% CI of the difference −9.8 to −0.7; *P* = .04) and oxygen saturation higher (97% vs 94%; mean difference, 3%; 95% CI of the difference, 1.4-3.7; *P* < .001) with VHC-1.

**Conclusions and Relevance:**

In this study, young children with acute wheezing experienced greater clinically significant improvement when salbutamol was administered through a VHC with high in vitro drug delivery compared with low in vitro drug delivery. This indicates that device choice matters and that guidelines should provide device-specific recommendations for pediatric inhalation therapy.

**Trial Registration:**

ClinicalTrials.gov Identifier: NCT03900494

## Key Points

**Question:**

Among children aged 6 to 48 months with acute wheezing, does a valved holding chamber (VHC) with higher in vitro drug delivery provide superior clinical effectiveness compared with a lower-delivery VHC?

**Findings:**

In a multicenter randomized clinical trial of 80 children across 4 emergency departments, children aged 6 to 48 months treated with the higher-delivery VHC had lower posttreatment respiratory distress assessment instrument scores than those treated with the lower-delivery VHC (2.7 vs 6.8) and a lower hospitalization rate (20% vs 50%).

**Meaning:**

In this study, a higher drug-delivery VHC yielded better clinical response, indicating that device choice matters and that guidelines should provide device-specific recommendations for pediatric inhalation therapy.

## Teaser

This randomized clinical trial attempts to determine whether a higher-delivery valved holding chamber improves clinical outcomes of salbutamol treatment compared with a lower-delivery valved holding chamber in young children with acute wheezing.

## Introduction

Acute wheezing accounts for approximately 10% of emergency visits in young children, resulting in hospitalization in 30% to 50% of cases.^1^ Bronchial obstruction is commonly treated with inhaled salbutamol (also known as albuterol), administered using pressurized metered-dose inhalers (pMDIs) through valved holding chambers (VHCs), a method considered at least as effective as the use of nebulizers in this context.^1,2^

Although commercially available VHCs used in pediatric inhalation therapy are often considered interchangeable, they vary substantially in material, electrostatic properties, internal volume, dead space, and valve design.^3,4,5,6,7,8,9,10,11^ Aerosol delivery performance of VHC–mask combinations varies widely; different devices paired with the same pMDI can result in marked differences in measured drug output,^3,4,12,13,14,15,16,17,18,19^ particularly under simulated conditions mimicking acute wheezing in children younger than 4 years.^16,18,19^ Despite these well-documented in vitro differences, evidence on the clinical impact of different commercially available VHCs in pediatric inhalation therapy remains limited. Most pediatric clinical studies have compared improvised valveless spacers with commercial VHCs in children 3 to 5 years old.^20,21,22,23,24,25,26,27^ Few trials have directly compared different commercial VHC models.^28,29,30,31,32^ Critically, there are currently no randomized clinical trials evaluating the clinical efficacy of different commercial VHCs for salbutamol treatment in young children with acute wheezing—one of the most common diagnoses in pediatric acute care.

In vitro studies show up to 23-fold differences in drug delivery between commercially available VHCs.^3,4,16,18,19^ Therefore, we hypothesized that the lower-delivery VHC would produce a less favorable clinical response than the higher-delivery VHC in young children with acute wheezing.

## Methods

### Trial Design and Oversight

**Figure 1.**
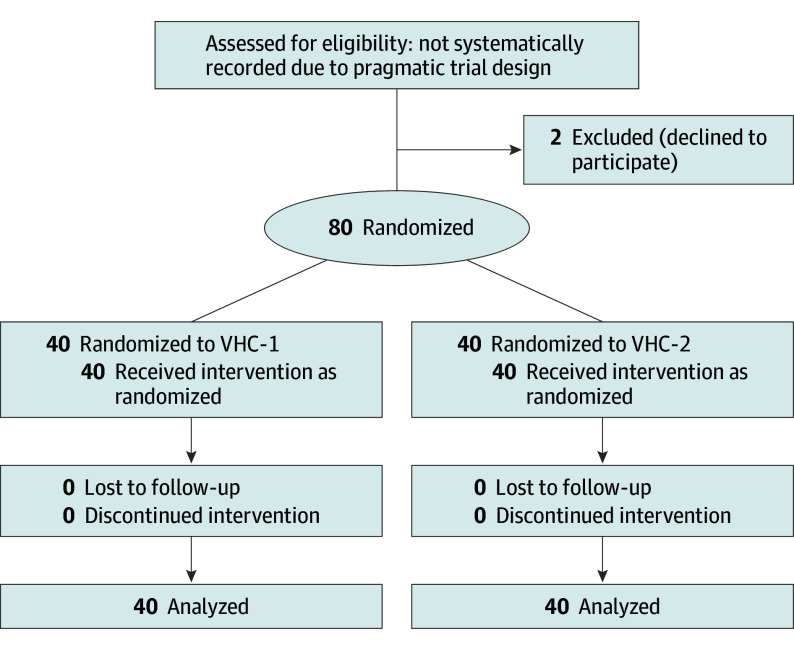
CONSORT Flow Diagram Showing Study Design Valved holding chamber group 1 (VHC-1) received salbutamol through a VHC with high drug delivery in vitro and VHC group 2 (VHC-2) received treatment through a VHC with low drug delivery in vitro.

We conducted an investigator-initiated, multicenter, randomized clinical trial (Figure 1) to compare 2 commercially available VHCs for salbutamol treatment in young children with acute wheezing: Optichamber Diamond (Philips Respironics) (VHC group 1 [VHC-1]) and Babyhaler (GSK)(VHC group 2 [VHC-2]). Each VHC was used with its respective mask. The study took place at 3 pediatric emergency departments (ED) in university hospitals (Tampere, Oulu, and Kuopio) and 1 primary care clinic (Terveystalo Hospital Tampere) in Finland. The study was registered at ClinicalTrials.gov (NCT03900494) before patient enrollment began. This report follows the Consolidated Standards of Reporting Trials (CONSORT) reporting guidelines.

### Ethical Considerations

The trial was conducted in accordance with the principles of the Declaration of Helsinki and the International Council for Harmonization Good Clinical Practice guidelines. The study protocol was approved by the Regional Ethics Committee of Tampere University Hospital (Finland) on March 18, 2019 (decision number: R19030). The investigators had full access to the data and vouch for the integrity of the data analysis.

### Study Population

Eligible participants were children aged 6 to 48 months presenting with moderate or severe acute wheezing (Respiratory Distress Assessment Instrument [RDAI] score ≥6) (eTable 1 in Supplement 1)^33,34^ and whose guardians provided written informed consent. Exclusion criteria included the need for immediate inpatient care, peripheral oxygen saturation (SpO_2_) below 85% upon arrival, a diagnosis of bacterial or viral pneumonia, acute laryngitis (croup), suspected or confirmed foreign body in the airway, or the presence of inspiratory crackles, previously diagnosed liver or kidney dysfunction, being immunocompromised or having another chronic condition deemed exclusionary by the attending physician, bronchopulmonary dysplasia, current use of a long-acting β-agonist, previous participation in this study, refusal to take medication via VHC, or participation in another clinical trial within 30 days before enrollment. Study participants were enrolled from April 17, 2019, through May 23, 2025. Enrollment was interrupted between March 2020 and July 2022 due to restrictions imposed by Finnish hospitals and research institutions during the COVID-19 pandemic on patient enrollment for non–COVID-19 clinical trials.^35^

### Randomization

Children were randomized 1:1 using a center-specific, computer-generated allocation list prepared by an independent biostatistician not involved in data collection. Permuted block randomization with varying block sizes (2, 4, or 6) was used. After written informed consent was obtained from the child’s guardian, each enrolled child received the next sequential study number. The corresponding opaque, sealed envelope was opened by the nurse to reveal the allocated VHC for salbutamol treatment.

### Blinding

Blinding of the treatment assignment was maintained throughout the study for physicians treating the patient and evaluating the treatment response. Inhalation therapy was administered by a nurse in a separate room, ensuring that the treating physician had no visual contact with the VHC used. Given the distinct physical appearance of the 2 VHCs, the nurse, guardians, and child were not blinded. This was necessary because correct treatment administration requires the nurse to visually monitor the spacer’s use, including mask fit and the valve movement in synchrony with the child’s breathing. The treatment codes remained blinded to investigators until all study data had been collected. Analyses were performed using group codes and the device identities were revealed only upon completion of the final analysis.

### Interventions

Two commercially available VHCs were selected for comparison: VHC-1 (Optichamber Diamond) had shown the highest drug delivery performance under simulated in vitro conditions for acute wheezing in young children, while VHC-2 (Babyhaler) had demonstrated markedly lower drug output in vitro but is the VHC most commonly used in Finland.^3,11,16,19^ Both VHCs are CE-marked and commercially distributed across multiple countries.

**Figure 2.**
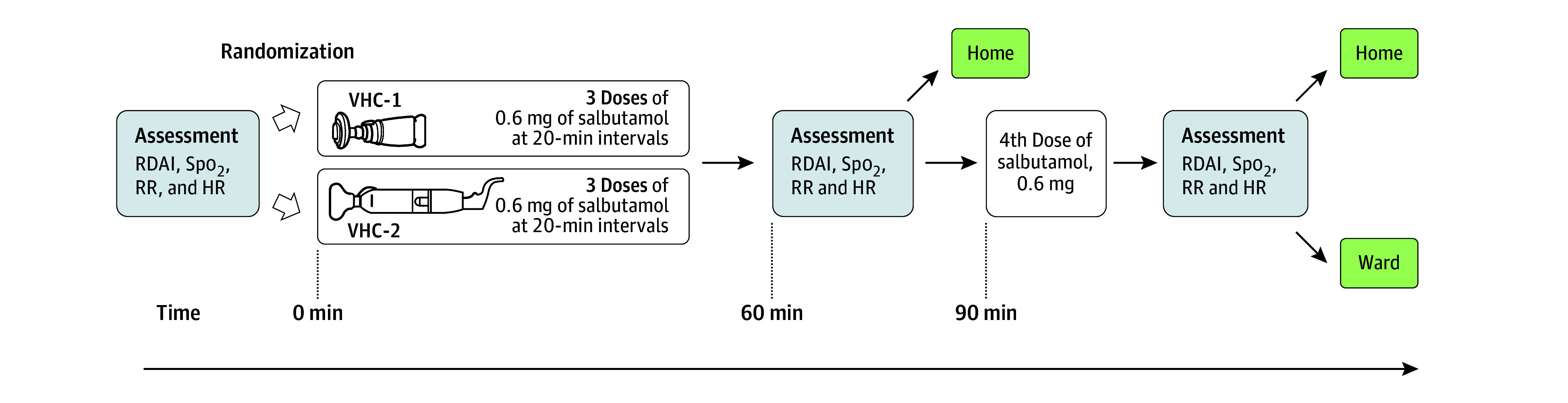
Timeline of the Study in Pediatric Acute Care Comparing 2 Commercially Available Valved Holding Chambers (VHCs) In previous studies, VHC group 1 (VHC-1) had high drug output in vitro and VHC group 2 (VHC-2) had low drug output in vitro. HR indicates heart rate; RDAI, respiratory distress assessment instrument; RR, respiratory rate; SpO_2_, oxygen saturation.

Each child received salbutamol (Ventoline Evohaler, 100 μg per puff [GSK]) via 1 of the 2 study VHCs with masks (eFigure 1 and eTable 2 in Supplement 1). For children weighing less than 25 kg, the initial salbutamol dose was 0.6 mg (6 puffs of 100 μg) administered up to 3 times at 20-minute intervals, according to national guidelines (total dose of 1.8 mg).^6,36^ Salbutamol was administered 1 puff at a time into the VHC, followed by at least 5 tidal breaths through the device. The physician reassessed the child’s clinical status 20 minutes after the third dose to evaluate treatment response. If clinically necessary, a fourth salbutamol dose of 0.6 mg was administered, followed by a final assessment 20 minutes later. Based on clinical evaluation, the physician determined whether the child should be discharged or hospitalized (Figure 2). This regimen follows commonly used ED protocols for moderate to severe exacerbations treated with a pMDI plus VHC and is consistent with Global Initiative for Asthma recommendations.^6^ Electrocardiogram monitoring was not required for short-acting β2-agonist therapy at these doses. Pulse oximetry and vital signs were recorded at baseline and after each dosing interval. Study staff at all sites received standardized training.

The child’s cooperation during inhalation therapy was assessed by both the administering nurse and the child’s guardian (higher score indicates better cooperation) (eTable 3 in Supplement 1). If the cooperation score was 2 or less, the same dose was reattempted once. All subsequent clinical follow-up and care proceeded in accordance with standard clinical practice. Concomitant treatments, including oral corticosteroids and other supportive therapies, were permitted and administered at the treating physician’s discretion in accordance with routine emergency care.

### Outcome Measures

We selected the RDAI as the primary outcome because it is a clinically relevant and validated score that measures acute lower-airway obstruction in preschool children.^37^ RDAI is noninvasive, quickly repeatable at the bedside, and was assessed by physicians blinded to device assignment, minimizing measurement bias. Because salbutamol delivered via a VHC is intended to relieve airflow obstruction, RDAI, which comprises wheeze and retractions, provides a direct, examiner-based clinical measure of treatment effect. We compared the change in RDAI score from baseline and the proportion of children achieving an RDAI decrease of 2 points or more between the treatment groups.^38^

Secondary outcomes included the proportion of children hospitalized, the proportion of children requiring a fourth salbutamol dose in the ED, posttreatment respiratory rate (RR) and its difference from baseline, posttreatment oxygen saturation (SpO_2_) and its difference from the baseline, and posttreatment heart rate and its difference from the baseline. Total time spent in the ED, not prespecified in the protocol, was analyzed as an exploratory post hoc outcome.

### Sample Size

Based on previous studies,^33,34^ children with acute wheezing presenting to the ED had an estimated mean RDAI score of 11, which was expected to decrease to 5 following standard salbutamol treatment. A posttreatment difference of at least 2 RDAI points between groups was considered clinically significant.^38^ Assuming a standard deviation of 2.5, a 2-sided α of .05 and 90% power, 33 children per group were required. To account for a 15% loss to follow-up, the target enrollment was set at 40 participants per group, totaling 80 participants.

### Statistical Analysis

All statistical analyses were performed in the intention-to-treat population using predefined outcomes, comparing the VHC-1 and VHC-2 groups. The unpaired *t* test was used for continuous, normally distributed variables and the Mann-Whitney *U* test for nonnormally distributed data. The proportions were compared using standard normal deviate test (*z* test for proportions). We reported proportions, risk differences, risk ratios, and numbers needed to treat (NNT), all with 95% CIs. Missing data were minimal, as all children remained under observation during acute care. We conducted a predefined sensitivity analysis excluding children with poor adherence, defined as a mean cooperation score below 4 (eTable 3 in Supplement 1). Statistical significance (type I error) is given only for the primary outcome. Other predefined secondary outcomes have been presented using the 95% CIs of the difference which have not been adjusted for multiple comparisons.

A post hoc sensitivity analysis of the primary outcome was conducted using linear mixed-effects models that were adjusted for baseline RDAI. We did a similar sensitivity analysis based on the recruitment era (pre- vs post-COVID-19).

Statistical analyses were performed using IBM SPSS Statistics version 30.0 (IBM) and StatsDirect version 4.0.4 (StatsDirect) and were completed between May 28, 2025, and September 8, 2025.

## Results

### Patients

A total of 80 young children with acute wheezing were enrolled and randomly assigned in a 1:1 ratio to receive salbutamol treatment using VHC-1 (n = 40) or VHC-2 (n = 40) (Figure 1). Baseline characteristics were balanced between the groups (Table 1). At study entry, the mean (SD) RDAI scores were 11.3 (3.0) and 9.9 (3.0) in the VHC-1 and VHC-2 groups, respectively.

**Table 1.** Demographic and Clinical Characteristics of Patients at Baseline

Characteristics at study entry	No. (%)			
	VHC-1 (n = 40)^a^		VHC-2 (n = 40)^b^	
Sex				
Female	15	(38)	14	(35)
Male	25	(62)	26	(65)
Age, mo, mean (SD)	21.6	(9.7)	24.6	(11.0)
Atopic dermatitis	26	(40)	21	(53)
Allergies	7	(18)	11	(28)
No previous wheezing	20	(50)	19	(48)
Smokers in the household	9	(23)	11	(28)
Pets at home	22	(55)	18	(45)
Parental asthma	14	(38)	13	(35)
Parental allergy	18	(49)	17	(45)
Parental atopic dermatitis	12	(33)	17	(45)
Siblings with asthma^c^	6	(24)	5	(21)
Siblings with allergy^c^	7	(28)	4	(17)
Siblings with atopic dermatitis^c^	4	(16)	10	(42)
ICS during the previous 4 wk	2	(5)	3	(7.5)
Oral prednisolone prior to ED visit^d^	0	(0)	1	(2.5)
RDAI score at presentation, mean (SD)	11.3	(3.0)	9.9	(3.0)
Respiratory rate, mean (SD)	57	(12.0)	56	(14)
Oxygen saturation, mean (SD)	93	(2.5)	93	(1.9)
Heart rate, mean (SD)	148	(7.8)	142	(17.3)
Cooperation score below 4	10	(25.0)	23	(57.5)

Abbreviations: ED, emergency department; ICS, inhaled corticosteroid; RDAI, respiratory distress assessment Iinstrument; RR, respiratory rate; SpO_2_, oxygen saturation; VHC, valved holding chamber.

^a^ VHC group 1 (VHC-1) received salbutamol through a valved holding chamber with high drug delivery performance in vitro.

^b^ VHC group 2 (VHC-2) received salbutamol through a valved holding chamber with low drug delivery performance in vitro.

^c^ Number of children with siblings in VHC-1 group, n = 25 and in VHC-2, n = 24.

^d^ The proportion of children receiving oral corticosteroids within 2 days prior to ED visit.

### Primary Outcome

**Figure 3.**
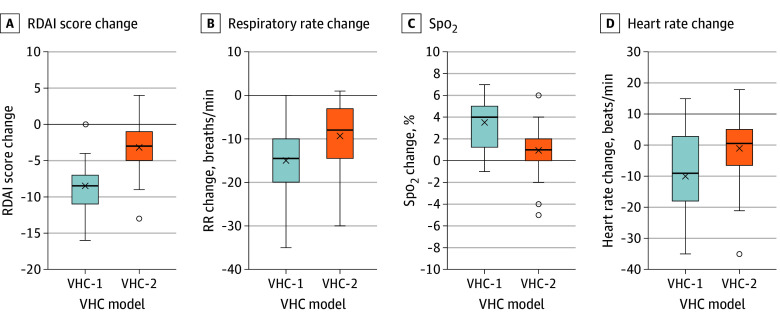
Clinical Response to Salbutamol in Young Children With Acute Wheezing According to the Used Valved Holding Chamber (VHC) VHC group 1 (VHC-1) had high drug output and VHC group 2 (VHC-2) group low drug output in vitro. Change from values measured at presentation to those measured postemergency department care are presented. X indicates the mean value, horizontal line the median value, and whiskers the range from the lowest to highest values within 1.5 × IQR. Small circles indicate the outliers. Differences between groups are statistically significant from A to C. RDAI indicates respiratory distress assessment instrument; SpO_2_, oxygen saturation.

Children treated with salbutamol using VHC-1 had lower respiratory distress as measured by the RDAI score after treatment than those treated using VHC-2 (Table 2; Figure 3; eFigure 2 in Supplement 1). The posttreatment mean (SD) RDAI score was significantly lower in the VHC-1 group (2.7 [2.1]) than in the VHC-2 group (6.8 [3.6]) with a mean difference of −4.1 (95% CI, −5.4 to −2.7).

**Table 2.** Primary and Secondary Outcomes of Young Children With Acute Wheezing Receiving Salbutamol Through 2 Commonly Used Commercial Used Valved Holding Chambers (VHCs)

Characteristic	VHC-1 (n = 40)	VHC-2 (n = 40)	RR (95% CI)	Between-group difference (95% CI)	NNT (95% CI)
Primary outcome					
Posttreatment RDAI, mean (95% CI)	2.7 (6.8-3.4)	6.8 (5.6-7.9)		−4.1 (−5.4 to −2.7)^a^	
RDAI change, mean (95% CI)	−8.6 (−9.5 to −7.6)	−3.2 (−4.3 to −2.0)		−5.4 (−6.9 to −3.9)^a^	
Decrease of RDAI score by ≥2, No. (%)	39 (98)	28 (70)	1.39 (1.17-1.79)	28 (14-44)^a^	3.6 (2.3-7.7)
Secondary outcomes, No. (%)					
Hospitalization	8 (20)	20 (50)	0.63 (0.43-0.87)	30 (9-49)	3.3 (2.1-11)
Need for 4th dose of salbutamol	23 (58)	32 (80)	0.72 (0.51-0.97)	22 (2-41)	4.4 (2.4-48)
Posttreatment RR, mean (95% CI)	42 (39-45)	47 (43-51)		−5.3 (−9.8 to −0.7)	
RR change, mean (95% CI)	−15 (−17 to −13)	−9.3 (−12 to −7.1)		−5.6 (−8.9 to −2.3)	
Posttreatment SpO_2_, mean (95% CI)	97 (96-98)	94 (93-95)		2.5 (1.4-3.7)	
SpO_2_ change, mean (95% CI)	3.5 (2.8-4.2)	0.9 (0.3-1.6)		2.6 (1.6-2.6)	
Posttreatment heart rate, mean (95% CI)	138 (131-144)	141 (135-146)		−3.1 (−12 to 5.7)	
Heart rate change, mean (95% CI)	−10 (−18 to −4)	−1 (−4 to 2)		−9.0 (−17 to 1.3)	

Abbreviations: ED, emergency department; NNT, number needed to treat; RDAI, Respiratory Distress Assessment Instrument score; RR, respiratory rate; SpO_2_, oxygen saturation; VHC-1, VHC group 1; VHC-2, VHC group 2.

^a^ *P* < .001.

The mean (SD) reduction in RDAI score from baseline was significantly greater in the VHC-1 group (−8.6 [3.0]) than in the VHC-2 group (−3.2 [3.7]) with a mean difference of −5.4 (95% CI, −6.9 to −3.9) (Figure 3). The proportion of children achieving an RDAI score decrease of 2 or more was 98% (39 of 40) with VHC-1 group vs 70% (28 of 40) with VHC-2 with an absolute risk difference of 28% (95% CI of the difference, 14%-44%), corresponding to an NNT of 3.6 (95% CI, 2.3-7.7).

### Secondary Outcomes

The proportion of children requiring hospitalization was significantly lower in the VHC-1 group (8 of 40 [20%]) than in the VHC-2 group (20 of 40 [50%]) with an absolute difference of 30% (95% CI of the difference, 9.2%-49%) corresponding to an NNT of 3.3 (95% CI, 2.1-11) to avoid 1 hospitalization (Table 2). The relative risk for hospitalization with VHC-1 compared with VHC-2 was 0.63 (95% CI, 0.43-0.87).

After salbutamol treatment, mean respiratory rate was lower in the VHC-1 group (42 vs 47 breaths per minute; mean difference, −5; 95% CI of the difference −9.8 to −0.7) and mean oxygen saturation was higher (97% vs 94%; mean difference, 3%; 95% CI of the difference, 1.4-3.7) (Table 2; Figure 3). The proportion of children requiring additional (fourth) salbutamol doses at the ED was lower in children treated with VHC-1 (58%) than with VHC-2 (80%), an absolute difference of 22% (95% CI of the difference 2.1% to 41%).

### Sensitivity Analysis

Per protocol, when the cooperation score was 2 or less, the same dose was reattempted once. As expected in this age group, reattempt success varied, but these reattempts did not change the study outcomes. In the sensitivity analysis excluding children with poor adherence (mean cooperation score below 4 [n = 47]), the between-group difference in mean change in RDAI score remained statistically significant at −5.5 (95% CI, −7.9 to −2.9).

### Exploratory Post Hoc Analyses

There was no significant difference in time spent in the ED: mean, 138 (95% CI, 127-151) minutes with VHC-1 vs 148 (95% CI, 137-159) minutes with VHC-2; mean difference, −10 minutes (95% CI, −26 to 7). During the ED visit, oral prednisolone was administered to 7 children (18%) in the VHC-1 and 8 children (20%) in the VHC-2 group. After adjustment for baseline severity, children treated with VHC-1 had lower posttreatment RDAI scores than those treated with VHC-2 (adjusted mean difference, −4.53 points; 95% CI, −5.80 to −3.26; *P* < .001). In sensitivity analysis adjusting for the recruitment period (pre– and post–COVID-19), the between-group difference in the primary outcome (posttreatment RDAI) remained statistically significant in both periods.

### Safety Outcomes

There was no statistically significant difference in heart rate after salbutamol treatment between groups (Table 2; Figure 3). No cardiovascular or any other severe adverse events were reported. None of the children required intensive care or died.

## Discussion

In this randomized clinical trial, young children with acute wheezing experienced significantly greater clinical improvement, as measured by RDAI scores, when salbutamol was administered through a VHC with high drug-delivery efficiency compared with one with substantially lower drug-delivery performance. Treatment with the higher-performing device was also associated with a lower risk of hospitalization. These findings support the use of a well-performing, commercially available VHC for the acute management of wheezing in young children.

The observed clinical differences are consistent with previous in vitro studies showing that VHCs differ markedly in drug-delivery efficiency, dependent on their design and the simulated breathing pattern.^15,16,19,39^ For instance, under conditions mimicking acute wheezing in young children, VHC-1 delivered 43 μg of a 100-μg salbutamol dose, whereas VHC-2 delivered only 1.8 μg.^3^ Thus, VHC-2 provides only a small fraction of the nominal dose, particularly when used with a face mask during respiratory distress.^3,16^ Our findings suggest that large in vitro performance differences of VHCs translate into significant differences in the clinical treatment response. Previous studies reporting no difference between spacer devices largely involved older children with stable asthma and relied on spirometry-based outcomes,^29,30^ which may not reflect the clinical response in young children with acute wheezing, as reported here.

The present findings have the potential to transform clinical practice. Although inhaled medications are strictly regulated, the devices used for their delivery, such as VHCs, are frequently considered interchangeable in both clinical guidelines and practice. In Finland, for instance, approximately 90% of EDs, including university hospitals, use a commercially available chamber (namely the VHC-2 of this study)^11^ shown to have suboptimal drug delivery under simulated in vitro breathing conditions mimicking acute wheezing in young children.^3,16^ The present results challenge this assumption, indicate that device selection can materially affect treatment efficacy in young children with acute wheezing.

Heart rate did not differ significantly between groups after the salbutamol treatment. Because heart rate is affected by multiple factors in the acute setting, it may not serve as a reliable indicator of systemic β_2_-agonist exposure. Nonetheless, VHC-1 achieved a greater clinical response at the same nominal salbutamol dose with no evidence of increased systemic adverse effects compared with VHC-2.^40^ These findings suggest that VHC-1 enhanced pulmonary delivery while remaining below the threshold for clinically relevant systemic stimulation.

### Strengths and Limitations

This study has several strengths. The clinical hypothesis was based on experimental in vitro evidence demonstrating clear differences in drug delivery between commercially available VHCs. Furthermore, the trial was conducted within routine pediatric emergency care without additional procedures or disruption to standard workflows, enhancing the generalizability of the findings. We enrolled young children with physician-diagnosed acute wheezing—the age group in which this condition is most common. The pragmatic design captured variability in cooperation and mask fit, both key determinants of VHC performance.^39,41^ Adjustment for baseline severity of respiratory distress did not change the findings. Wheezing severity was assessed using the validated RDAI score and clinical outcomes were derived from routinely monitored parameters in emergency care. No participants were lost to follow-up.

This study should be interpreted considering the following limitations. Blinding of the VHC for nurses and patients was not feasible, as visual monitoring was required for correct salbutamol administration. However, all primary outcomes were assessed by a blinded physician, which minimized potential assessment bias. The findings apply specifically to the 2 VHC models tested and should not be generalized to all commercially available devices. Although the trial used a multicenter design, all participants were recruited in Finland, which may limit generalizability to more diverse populations or low-income settings. There was no formal patient or caregiver involvement in the study design. However, a recent publication on outcome prioritization in preschool wheeze found symptom improvement and hospitalization as parent-important outcomes,^42^ which is consistent with our end point strategy. Because VHC-2 is the most used device locally, any familiarity bias could have favored it; yet results consistently favored VHC-1 and were unchanged after excluding children with suboptimal cooperation.

Regulatory pathways for VHCs prioritize safety and basic bench performance and do not require comparative clinical effectiveness in young children. Clearance typically relies on tests under steady-flow conditions whereas key determinants of delivered dose, such as electrostatics, dead space, mask leak/fit, are not uniformly specified or evaluated with pediatric tidal breathing. Furthermore, head-to-head equivalence is not mandated. This explains the substantial between-device variability despite regulatory compliance, consistent with the large in vitro differences and the clinical effects observed here. Our findings support standardized performance testing and comparative evidence to guide procurement and guideline recommendations for children.

## Conclusions

In conclusion, this trial demonstrates a significant difference in clinical response to salbutamol treatment between 2 commercially available VHCs in young children with acute wheezing. The results indicate that clinical guidelines must not assume interchangeability of VHC devices and highlight the need for device-specific recommendations in pediatric inhalation therapy.

## Notes

Supplement 1. eTable 1. Respiratory Distress Assessment Instrument (RDAI) scoring eTable 2. Valved holding chambers VHC-1, Optichamber Diamond and VHC-2, Babyhaler eTable 3. Assessment of co-operation during inhalation therapy using a structured scoring system eFigure 1. Valved holding chambers eFigure 2. Change in Respiratory Distress Assessment Instrument (RDAI) score from presentation to the emergency department (ED) to post-ED care in the VHC-1 and Babyhaler groups eFigure 3. Mean nurse-assessed co-operation score (from 0 to 5) by study unit and VHC model. Higher scores indicate better cooperation eReferences

Supplement 2. Statistical analysis report

Supplement 3. Data sharing statement
