# Supplementary material for: Valved Holding Chambers in Young Children With Acute Wheezing: A Randomized Clinical Trial
Source: JAMA Pediatr. 2026 Feb 23;180(5):567–74. doi: 10.1001/jamapediatrics.2025.6479 (PMC12931466; doi:10.1001/jamapediatrics.2025.6479)
Supplement: Supplement 1. — eTable 1. Respiratory Distress Assessment Instrument (RDAI) scoring eTable 2. Valved holding chambers VHC-1, Optichamber Diamond and VHC-2, Babyhaler eTable 3. Assessment of co-operation during inhalation therapy using a structured scoring system eFigure 1. Valved holding chambers eFigure 2. Change in Respiratory Distress Assessment Instrument (RDAI) score from presentation to the emergency department (ED) to post-ED care in the VHC-1 and Babyhaler groups eFigure 3. Mean nurse-assessed co-operation score (from 0 to 5) by study unit and VHC model. Higher scores indicate better cooperation eReferences [file jamapediatr-e256479-s001.pdf]

Supplemental Online Content

Csonka P, Ruuska-Loewald T, Hämynen I, et al. Valved holding chambers in young children with acute wheezing: a randomized clinical trial. *JAMA Pediatr*. Published online February 23, 2026. doi:10.1001/jamapediatrics.2025.6479

eTable 1. Respiratory Distress Assessment Instrument (RDAI) scoring

eTable 2. Valved holding chambers VHC-1, Optichamber Diamond and VHC-2, Babyhaler

eTable 3. Assessment of co-operation during inhalation therapy using a structured scoring system

eFigure 1. Valved holding chambers

eFigure 2. Change in Respiratory Distress Assessment Instrument (RDAI) score from presentation to the emergency department (ED) to post-ED care in the VHC-1 and Babyhaler groups

eFigure 3. Mean nurse-assessed co-operation score (from 0 to 5) by study unit and VHC model. Higher scores indicate better cooperation

eReferences

This supplemental material has been provided by the authors to give readers additional information about their work.

eTable 1. Respiratory Distress Assessment Instrument (RDAI) scoring[1]

	Points					Max. score
	0	1	2	3	4	
**Wheezing**						
Expiration	none	End	½	¾	all	4
Inspiration	none	Part	All	–	–	2
Lung fields	none	Part	All	–	–	2
**Retractions**						
Supraclavicular	none	Mild	Moderate	Marked	–	3
Intercostal	none	Mild	Moderate	Marked	–	3
Subcostal	none	Mild	Moderate	Marked	–	3

The maximum score for wheezing is 8, and for the use of accessory muscles 9.
Diminished breath sounds correspond to 8 points for wheezing.

eTable 2. Valved holding chambers VHC-1, Optichamber Diamond® and VHC-2, Babyhaler®. Price estimates are based on information provided by online retailers in Finland.

Valved holding chamber	Chamber volume	Price	Available masks
VHC-1	140 ml	25 €	Two pediatric face mask sizes, small for 0–18 months and medium for 1–5 year
VHC-2	350 ml	35 €	One size face mask

eTable 3. Assessment of co-operation during inhalation therapy using a structured scoring system

	Points					
Evaluated by the nurse	0	1	2	3	4	5
Evaluated by the parent/guardian	0	1	2	3	4	5

0 = medication administration failed; 1 = child is crying continuously; 2 = child is crying, screaming, or resisting, but not continuously; 3 = child shows some resistance to medication administration but does not cry or scream; 4 = good cooperation, but the child appears uncomfortable; 5 = good cooperation and calm breathing throughout medication administration. Adapted from Minh et al.[2] If the nurse rates the medication administration as 0–2 points, the same dose is repeated once.

© 2026 Csonka et al. *JAMA Pediatr*.

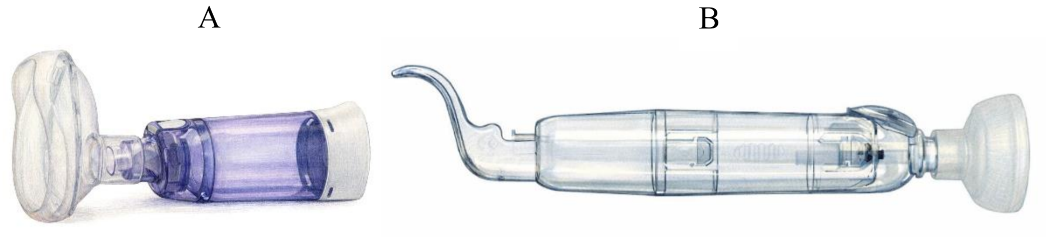

eFigure 1. Valved holding chambers. A) VHC-1, Optichamber Diamond®. B) VHC-2, Babyhaler®. Original illustration created by the authors; source photograph captured by the authors; the drawing was rendered from the photograph using a generative AI tool (OpenAI ChatGPT 5.2).

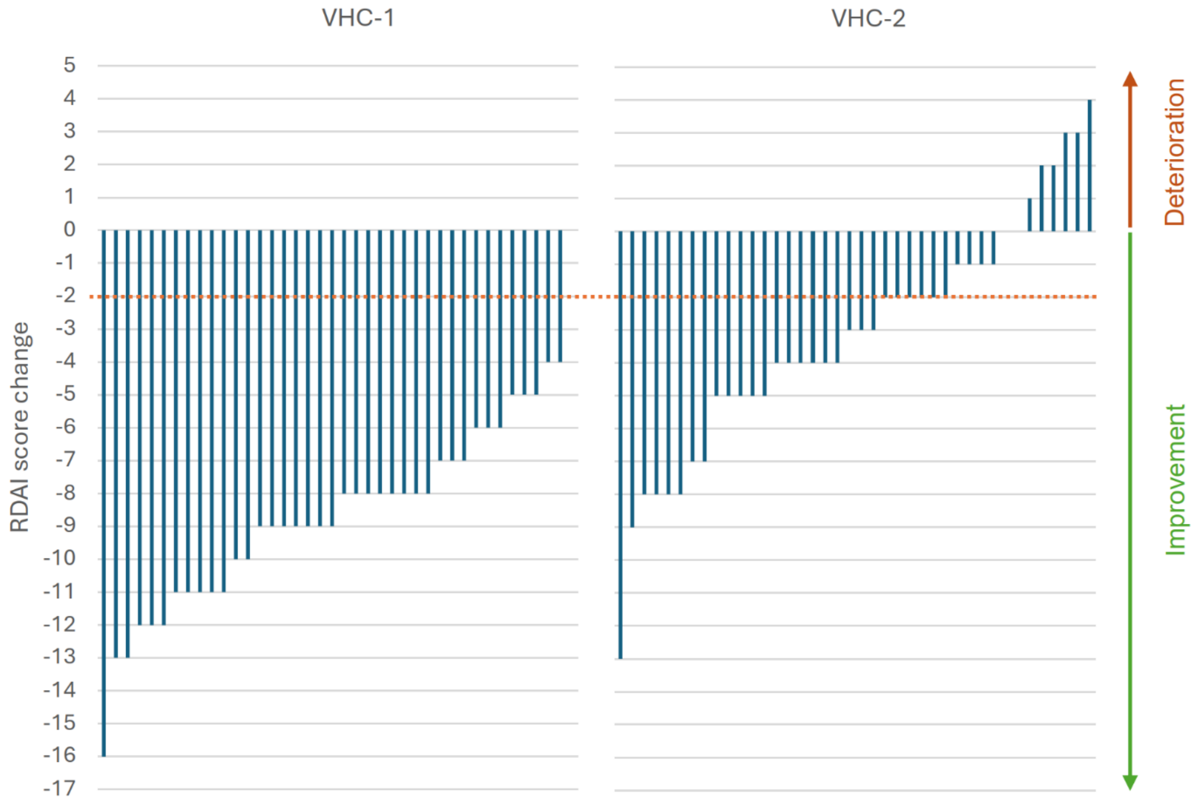

eFigure 2. Change in Respiratory Distress Assessment Instrument (RDAI) score from presentation to the emergency department (ED) to post-ED care in the VHC-1 and Babyhaler groups. Each bar represents an individual child's change in RDAI score between presentation and post-ED care. Lower values indicate greater improvement in respiratory symptoms. The dashed line denotes the threshold for clinically significant improvement.

© 2026 Csonka et al. *JAMA Pediatr*.

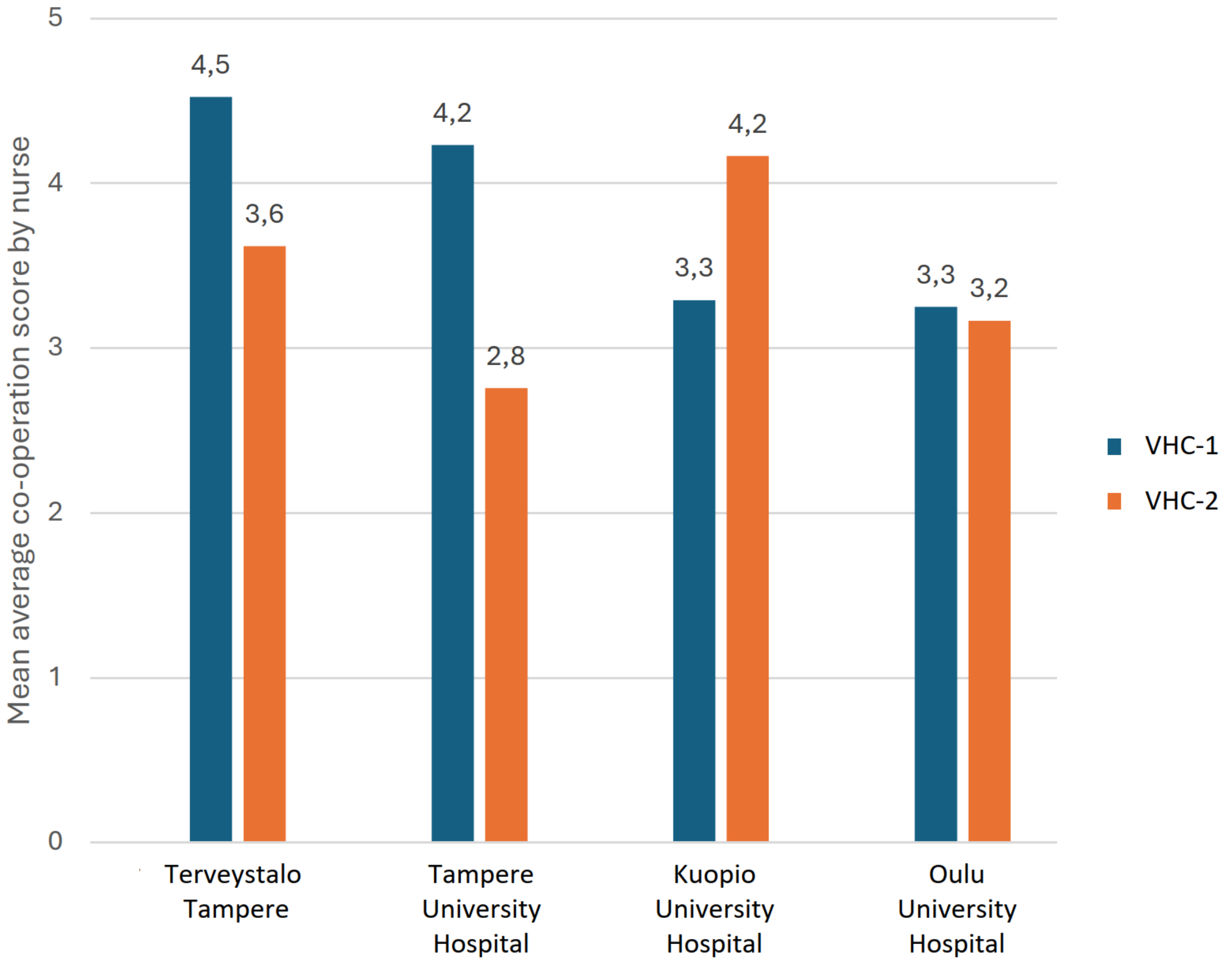

eFigure 3. Mean nurse-assessed co-operation score (from 0 to 5) by study unit and VHC model. Higher scores indicate better cooperation.

eReferences

1. Lowell DI, Lister G, Von Koss H, McCarthy P. Wheezing in Infants: The Response to Epinephrine. Pediatrics 1987;79(6):939–45.

2. Minh KT, von Hollen D, von Königslöw AJ, Nikander K, Janssens HM. An Instrumented Valved Holding Chamber with Facemask to Measure Application Forces and Flow in Young Asthmatic Children. Journal of Aerosol Medicine and Pulmonary Drug Delivery 2014;27(S1):S-55-S-62.

© 2026 Csonka et al. *JAMA Pediatr*.
