# Supplementary material for: Valved Holding Chambers in Young Children With Acute Wheezing: A Randomized Clinical Trial
Source: JAMA Pediatr. 2026 Feb 23;180(5):567–74. doi: 10.1001/jamapediatrics.2025.6479 (PMC12931466; doi:10.1001/jamapediatrics.2025.6479)
Supplement: Supplement 2. — Statistical analysis report [file jamapediatr-e256479-s002.pdf]

Study protocol for the manuscript

Valved Holding Chambers in Young Children with Acute Wheezing – A Randomized Clinical Trial

The file contains the original study protocol, including the statistical analysis plan. The protocol was amended twice, on February 25, 2021, and on January 13, 2025 with all amendments marked in red on page 3, 7, and 12.

Finnish-language Case Report Forms and the Informed Consent Form can be provided upon request.

CHAMBER study

Comparing the efficacy of two valved holding chambers for bronchodilator administration in 0.5-4 years old children with acute wheezing – a randomized clinical trial

Principal investigator
Péter Csonka, MD, PhD, Associate professor in pediatrics, TamCAM - Tampere Center for Child, Adolescent and Maternal Health Research, Tampere University and Terveystalo Healthcare, Finland

Group
Sauli Palmu, MD, PhD, Associate professor in pediatrics, TamCAM - Tampere Center for Child, Adolescent and Maternal Health Research, Tampere University and Tampere University Hospital, Finland

Lauri Lehtimäki, MD, PhD, Professor of respiratory medicine, Tampere University Hospital

Balázs Kelemen, MD, Tampere University Hospital, Finland

Salla Kuusela, MD, Tampere University Hospital, Finland

Iida Ojaniemi, MD, Tampere University Hospital, Finland

Marjo Renko, MD, PhD, Professor in pediatrics, Kuopio University Hospital, Finland

Inka Hämynen, MD, Kuopio University Hospital, Finland

Eeva Mykkänen, MD, Kuopio University Hospital, Finland

Terhi Tapiainen, MD, PhD, Professor in pediatrics, Oulu University Hospital, Finland

Minna Honkila, MD, PhD, Oulu University Hospital, Finland

Minna Juntunen, MD, Oulu University Hospital, Finland

Protocol 19.3.2019 - Amended in 25.2.2021 - Amended in 11.1.2025.

Summary

Approximately 4–7% of children have asthma requiring regular medication. In addition, an equally large proportion of children experience wheezing episodes triggered by infections. Among all pediatric emergency department visits, about 10% are due to respiratory distress, and up to 40% of these cases lead to hospitalization. Recurrent episodes of respiratory distress affect about 10% of children.

Acute respiratory distress is primarily treated with inhaled salbutamol, a bronchodilator, which is usually administered via a valved holding chamber (VHC). However, there are currently no recommendations on the optimal size or model of spacer device to be used in the treatment of acute respiratory distress. In a recent in vitro study conducted in Finland, significant differences were reported between commercially available VHCs in terms of their functional performance. Among these, the OptiChamber Diamond device showed moderately reliable performance. In contrast, the Babyhaler, currently the most widely used spacer in Finland, demonstrated notably low delivery efficiency, particularly at inspiratory flows typical of young children. The measured differences between devices were so substantial that they are presumed to influence clinical treatment efficacy, especially in acute cases.

The aim of this study is to determine whether the differences observed in *in vitro* performance between spacer devices are clinically relevant in vivo during emergency treatment. Specifically, the study will assess whether there is a difference between the Babyhaler and the OptiChamber Diamond in the treatment of acute respiratory distress in children in terms of:

1. symptom relief
2. likelihood of hospitalization

3. ~~recurrence of symptoms~~ *(Amended in 25.2.2021. Outcome removed because it was not feasible to collect the data reliably in all the centers)*
4. treatment acceptance
5. adverse effects.

This is a pragmatic randomized, investigator-blinded, multicenter clinical trial conducted in the pediatric emergency departments of Tampere, Oulu, and Kuopio University Hospitals, as well as at the Terveystalo outpatient clinic in Tampere. The study involves standard clinical care, with no blood samples or additional diagnostic procedures. Both VHCs are approved for pediatric use in Finland and internationally. Salbutamol treatment will follow national evidence-based care guidelines (Käypä hoito).

Based on sample size calculations, the study will include 80 children aged 6 months to 4 years who are diagnosed with moderate or severe bronchial obstruction. In the emergency department, the physician-investigator will assess the child's respiratory distress using a standardized clinical scoring tool. Each child will receive salbutamol (0.6 mg or 0.8 mg, based on weight) up to four times at 20-minute intervals via one of the two VHCs. The investigator will assess clinical response at scheduled intervals and determine whether the child should be discharged or admitted for inpatient care. Other aspects of follow-up and treatment will proceed according to standard clinical protocols.

Randomization lists for each site will be created using block randomization with randomly permuted block sizes of 2, 4, and 6. Each child will have an equal chance of being allocated to either VHC group.

Spacer devices are currently used with the assumption that they are interchangeable. However, recent *in vitro* studies suggest that the devices used in Finland may have substantial differences in drug delivery efficiency. One of the main goals of the Finnish National Allergy Program 2008–2018 was to focus efforts on preventing severe symptoms and asthma exacerbations. Respiratory symptoms related to airway obstruction remain a common issue in children, and reducing their frequency and effectively managing acute episodes is key to minimizing both patient suffering and healthcare costs. It is especially important to evaluate, standardize, and optimize critical treatment practices.

The regional ethics committee of the Tampere University Hospital special responsibility area has approved the study on the 18th of March 2019 (Ethics Committee Code: R19030). Recruitment is expected to begin in spring 2019. The results will be published in both Finnish and international peer-reviewed journals.

Introduction

Prevalence of Asthma and Asthma-like Symptoms in Children

Asthma is the most common chronic disease in children that qualifies for special reimbursement by the Finnish Social Insurance Institution (Kela). Approximately 4–7% of children have asthma requiring regular pharmacological treatment. In addition, an equally large proportion of children experience wheezing triggered by infections [1]. Among infants and preschool-aged children, 20–30% experience wheezing at least once during the first few years of life [2].

Children with infection-triggered wheezing, i.e., obstructive bronchitis, place a significant burden on hospitals, and those managed at home lead to significant loss of workdays for parents. Approximately 10% of all visits to pediatric emergency departments are due to respiratory distress, and up to 40% of these cases result in hospitalization [1]. Up to 80% of all return visits to pediatric emergency departments are due to respiratory distress (Csonka, unpublished data). Recurrent episodes of respiratory distress affect about 10% of children.

Emergency Treatment of Infection-Triggered Wheezing (Obstruction) with Inhaled Medications

Acute respiratory distress is primarily treated with inhaled salbutamol, a bronchodilator that relieves bronchial smooth muscle constriction. It is administered either via a valved holding chamber (Figure 1) or by nebulizer. Clinical studies have shown no significant difference in treatment efficacy between spacer and nebulizers in the treatment of acute asthma attacks in either adults or children [3-7].

In Finland, spacers are now used as the first-line delivery method in the acute treatment of bronchial obstruction. The medication used is salbutamol in metered-dose inhaler form (0.1 mg per actuation). According to the national evidence-based guideline (Käypä hoito), salbutamol is administered initially at a dose of 4–8 actuations (0.4–0.8 mg) every 20 minutes for 3–4 doses to reverse marked bronchoconstriction, followed by 2–4 actuations every four hours [8].

Figure 1. A) In children under the age of 3, inhaled medications are administered via pressurized metered-dose inhaler (pMDI) using a valved holding chamber with a face mask. Shown: OptiChamber Diamond (image © Philips). B) Around the age of 3, most children learn to seal the mouthpiece of the holding chamber tightly with their lips, allowing the mask to be discontinued. Shown: Babyhaler (image © P. Csonka).

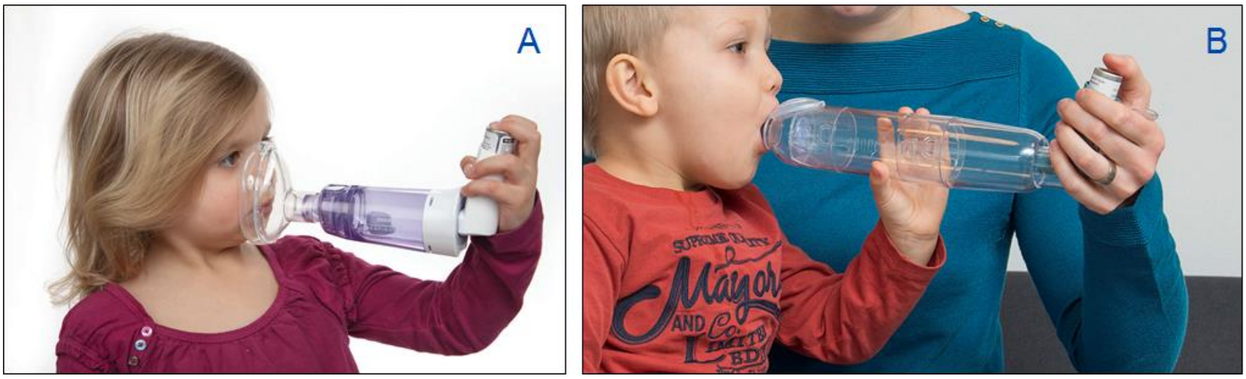

Use of Spacer Devices in Finland

Several different models of spacer devices and masks are available on the Finnish market (Table 1). There are no clear recommendations regarding which spacer should be used in the treatment of acute respiratory distress. According to a recent survey (Csonka & Lehtimäki, unpublished data), the Babyhaler brand spacer (Figure 1B and Figure 2) is predominantly used in hospitals and health centers across Finland.

Table 1. Spacer devices intended for pediatric use currently available on the Finnish market. Price estimates are based on information provided by online retailers.

Spacer	Chamber volume	Face mask volume	Price	Huomioitavaa
A2A Spacer	210 ml	80 ml	26 €	One size face mask
Aerochamber Plus	149 ml	35 ml	50 €	Four models, one without a mask, and three different mask sizes
Babyhaler	350 ml	80 ml	35 €	One size face mask
OptiChamber Diamond	140 ml	45 ml	25 €	Three face mask sizes
Vortex	194 ml	46 ml	45 €	Two face mask sizes

Figure 2. Spacer devices used in hospital and health center emergency departments. A nationwide survey (Csonka & Lehtimäki) is currently ongoing, with responses received from 51 out of 100 units across Finland to date. In some units, multiple devices may be used interchangeably. The Volumatic device is not intended for use in children.

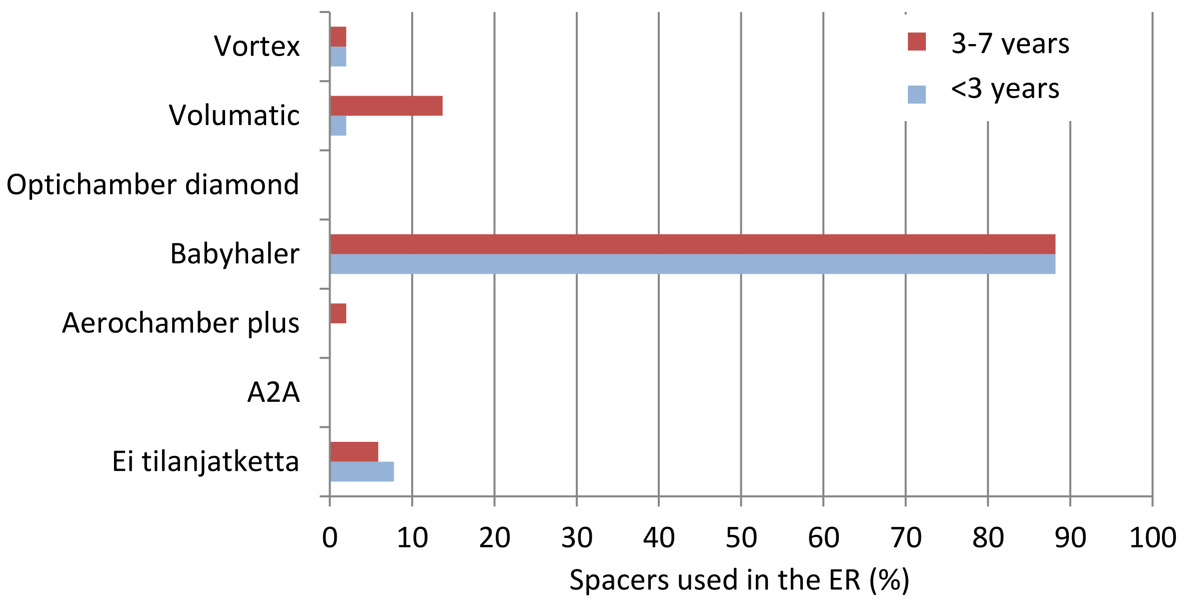

Spacer Device Characteristics and Differences

The most important properties of a spacer device that significantly influence the effectiveness of inhaled medication delivery are:

1. the volume of the chamber
2. electrostatic charge
3. the shape and particle size of the aerosol plume
4. valve characteristics
5. mask properties.

In a recent experimental study, the delivery efficiency (fine particle dose) of spacer devices currently available on the Finnish market (Table 1) was measured under standardized in vitro conditions using various breathing rates and inspiratory volumes. Drug delivery efficiency was found to be strongly influenced by the child's breathing pattern and the use of a face mask (Csonka & Lehtimäki, *ERJ Open Res*, in press). In addition, the results demonstrated significant manufacturer-specific differences in the physical characteristics of the devices and, in particular, in the delivery efficiency of salbutamol. For some spacer models, the median delivered dose of salbutamol was as low as less than 1% of the nominal dose (Figure 3). Of the tested devices, OptiChamber Diamond and AeroChamber Plus showed moderately reliable performance both with and without a mask. The Babyhaler, by far the most widely used spacer in Finland, had low delivery efficiency (2.6% without a mask and 0.8% with a mask, relative to the nominal dose).

As early as 1999, Turpeinen et al. [9] reported that the amount of drug reaching the airways through devices from different manufacturers can vary by several-fold. Our own findings confirm earlier observations that spacer devices used for drug delivery should not be considered interchangeable, even if they appear similar externally. Differences between spacers and masks may significantly impact clinical outcomes and could potentially influence the frequency of emergency department visits and hospital

admissions. Each device's performance and usability in pediatric drug delivery must be evaluated individually [10].

Clinical Problem and Research Question

In studies comparing the effectiveness of spacers and nebulizers in the treatment of acute respiratory distress, a wide variety of spacer devices, both commercially manufactured and custom-made, have been used. Some studies fail to report the type of spacer used at all [3][7]. The efficacy of different spacer devices has not been systematically compared, and no comprehensive, independent (non-industry-funded) studies on this topic have been published. In Finland, national care guidelines (e.g., those available through the Terveysportti medical database) refer to spacers in general terms without addressing specific device characteristics or potential differences that could affect treatment outcomes.

To justify changes to clinical guidelines, *in vitro* findings must be supported by *in vivo* evidence demonstrating significant differences in treatment efficacy. For this study, we selected for comparison the Babyhaler (Figure 1B), currently the most commonly used spacer in Finnish emergency departments, and the OptiChamber Diamond (Figure 1A), which performed well in *in vitro* measurements and is also the most cost-effective option.

Study Objectives

The objective of this study is to determine whether the device differences observed in in vitro measurements have a clinically meaningful impact on treatment success in the emergency setting (in vivo). The differences in delivered drug dose between the Babyhaler (BH) and OptiChamber Diamond (OD) devices are substantial (Figure 3). These differences are assumed to affect the bronchodilator treatment response, particularly in young children with moderate to severe respiratory distress. In this patient group, breathing is often highly variable, shallow, and rapid—factors that make the administration of inhaled medications especially challenging.

The study will assess whether there is a difference between the Babyhaler and OptiChamber Diamond in the treatment of acute respiratory distress in children in terms of:

1. Symptom relief (Tables 2 and 3)
2. Probability of hospitalization
3. Symptom recurrence (return visits within 48 hours of discharge)
4. Treatment acceptance (which device children tolerate better; Table 4)
5. Adverse effects

Table 2. Respiratory Distress Assessment Instrument (RDAI) scoring [11]

	Points					Max. score
	0	1	2	3	4	
**Wheezing**						
Expiration	none	End	½	¾	all	4
Inspiration	none	Part	All	–	–	2
Lung fields	none	Part	All	–	–	2
**Retractions**						
Supraclavicular	none	Mild	Moderate	Marked	–	3
Intercostal	none	Mild	Moderate	Marked	–	3
Subcostal	none	Mild	Moderate	Marked	–	3

The maximum score for wheezing is 8, and for the use of accessory muscles 9.
Diminished breath sounds correspond to 8 points for wheezing.

Table 3. Clinical variables monitored and differences between groups

Main outcomes	Secondary outcomes
RDAI-scores<br>~~SaO2~~	SaO2 (*Amended in 13.1.2025. Decision made to have only one primary outcome that was the base of the sample size calculation. SaO2 was moved from Main outcomes to Secondary outcomes)*
	Change in respiratory rate (difference before and after medication)
	Heart rate (difference before and after medication)
	~~Need for oral prednisolone~~ *(Amended in 25.2.2021. The proportion of children receiving oral corticosteroids will be reported as baseline characteristic instead of outcome because they were often given before and/or during the intervention)*
	Number (proportion) of patients admitted to the hospital
	Parents' assessment of treatment success (Table 4)
	Healthcare personnel's assessment of treatment success (Table 4)
	Proportion of patients requiring a fourth dose of medication
	~~Number of return visits~~ *(Amended in 25.2.2021. Outcome removed because it is not feasible to collect the data reliably in all the centers)*

Table 4. Assessment of treatment adherence and perceived effectiveness of inhalation therapy using a structured scoring system

	Points					
Evaluated by the nurse	0	1	2	3	4	5
Evaluated by the parent/guardian	0	1	2	3	4	5

0 = medication administration failed; 1 = child is crying continuously; 2 = child is crying, screaming, or resisting, but not continuously; 3 = child shows some resistance to medication administration but does not cry or scream; 4 = good cooperation, but the child appears uncomfortable; 5 = good cooperation and calm breathing throughout medication administration. Adapted from Minh et al. [11]. If the nurse rates the medication administration as 0–2 points, the same dose is repeated once.

Study Implementation

Study Design

This study is a randomized, investigator-blinded, multicenter clinical trial conducted in the pediatric emergency departments of Tampere, Oulu, and Kuopio University Hospitals, as well as at the Terveystalo outpatient clinic on Rautatienkatu in Tampere, Finland. This is a pragmatic therapeutic clinical trial that does not involve blood sampling or any additional diagnostic procedures. Blinding is maintained for physicians involved in the emergency care inhalation therapy and research, for those entering data into the study database, and for the analysts evaluating the study results. The parents, child, and nurse are not blinded, as correct treatment administration requires the nurse to visually monitor the use of the spacer, including the fit of the mask and the movement of the valves in synchrony with breathing. The spacer devices being studied are approved for use in children both in Finland and internationally. The medication administered via the spacer follows the national evidence-based care guideline (Käypä Hoito) [8].

Study Population

The study will include a total of 80 children aged 6 months to 4 years who are diagnosed with moderate or severe bronchial obstruction (RDAI score ≥6, Table 2), and whose guardians have provided written, informed consent. The severity of respiratory distress is assessed using a previously published Respiratory Distress Assessment Instrument (RDAI) [12, 13].

Children will be excluded from the study at the emergency department if they meet any of the following criteria:

1. Require immediate inpatient care
2. Have an oxygen saturation (SpO_2) below 85% upon arrival
3. Are diagnosed with bacterial or viral pneumonia
4. Show inspiratory crackles (suggestive of bronchiolitis in infants)
5. Are diagnosed with acute laryngitis (croup)
6. Are suspected to have or are confirmed to have a foreign body in the airway
7. Have liver or kidney dysfunction
8. Are immunocompromised or have another chronic condition deemed exclusionary by the attending physician
9. Have bronchopulmonary dysplasia due to prematurity
10. Are currently using a long-acting beta-agonist (LABA)
11. Have previously participated in this study
12. Refuse to take medication via spacer
13. Have participated in another clinical trial within the 30 days prior to enrollment

Study Procedure from the Patient's Perspective

Recruitment and Randomization

When a child aged 6 months to 4 years presents to the emergency department with acute bronchial obstruction, it is first confirmed that the child does not require immediate hospitalization and does not have any of the previously listed exclusion criteria. If eligible, the purpose of the study is explained to the parents, and they are given a patient information sheet to read (Appendix X, which they may keep).

While the parents review the form, the emergency nurse notifies the study unit's investigator, who confirms the diagnosis and the need for treatment. If the investigator is on call, they may personally enroll the child into the study. If the parents decide to participate, they are asked to sign an informed consent form (Appendix Y), of which a copy is also provided to them.

Randomization is conducted by biostatistician Tytti Pokka (Oulu University Hospital), who is not involved in data collection. First, the spacer devices are coded as either Spacer A or Spacer B. Each center uses block randomization with randomly varying block sizes of 2, 4, or 6 (permuted blocks), as the study is single-blinded (blinding applies only to the study physician).

Randomization to the intervention or control group is carried out by assigning the first available study number between 001–00X to the child. Each participant has an equal probability of being allocated to either spacer group, as the assignment for each study number has been randomly pre-determined per center.

The assigned treatment group is sealed in an opaque envelope, which is opened in numerical order only after written consent has been obtained. The envelopes are prepared by a study nurse who does not participate in data collection. The group codes will not be unblinded until all study-related data has been recorded. Data analysis will be performed using the A and B group codes, meaning the investigators will not know which spacer corresponds to A or B until after the final analysis.

Treatment of the Enrolled Child in the Emergency Department

The study physician assesses the severity of the child's respiratory distress and records the RDAI score as well as other clinical variables listed in Table 3 on the study form. The child receives salbutamol via either the BH or OD spacer, with a dose based on weight (0.6 mg or 0.8 mg, corresponding to 6 or 8 actuations), up to four times at 20-minute intervals (Figure 4).

The study physician reassesses the clinical variables (Table 3) before treatment and again 20 minutes after the first two doses. If the child still requires bronchodilator treatment, up to two additional doses (0.6 or 0.8 mg, depending on weight) are administered. The physician again assesses breathing effort 20 minutes after the third and, if given, the fourth dose, and then makes the decision regarding further treatment (discharge or hospitalization, Figure 4). Otherwise, the follow-up and care of study participants will proceed in the outpatient clinic according to standard clinical practice.

In children under the age of 3, the spacer is used with a face mask; in older children, it may be used without a mask when appropriate. Salbutamol is administered one puff at a time into the spacer, and after each actuation, the child should take at least five breaths through the device. If the child strongly resists medication administration or is crying (Table 4), the same dose should be reattempted once the child has calmed down.

If a child enrolled in the Chamber study is discharged from the outpatient clinic, they will be given the spacer used during the study free of charge, along with written instructions for continued care. If the child is admitted to the hospital, treatment will continue on the ward using the same spacer device that was used in the emergency department.

Figure 3. Median dose of salbutamol delivered from spacer devices (% of nominal dose) and range measured using a filter (in vitro filter dose), when used without a mask (no mask) or with a mask (with mask). Measurements were performed using devices from three different production batches (LOT).

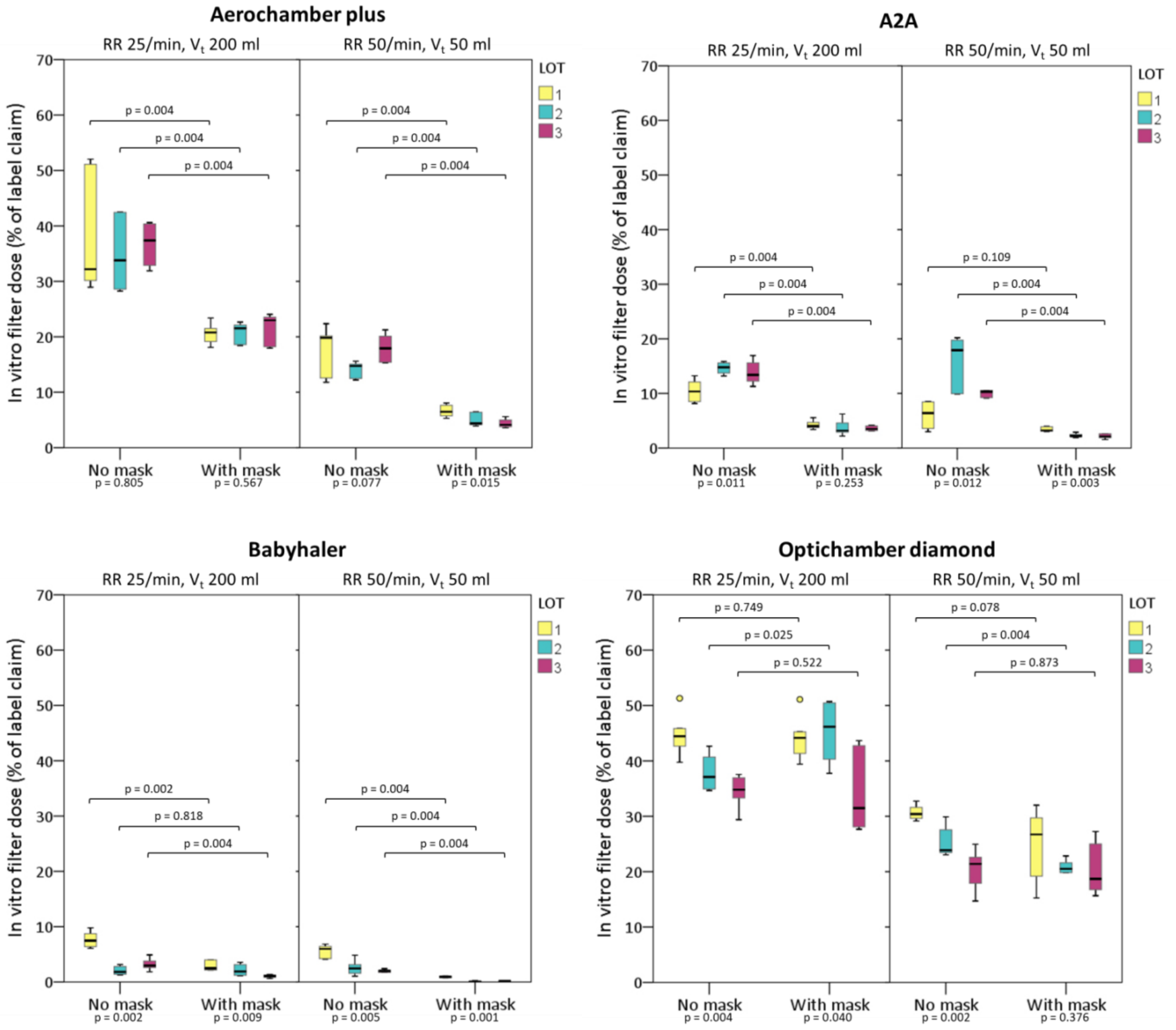

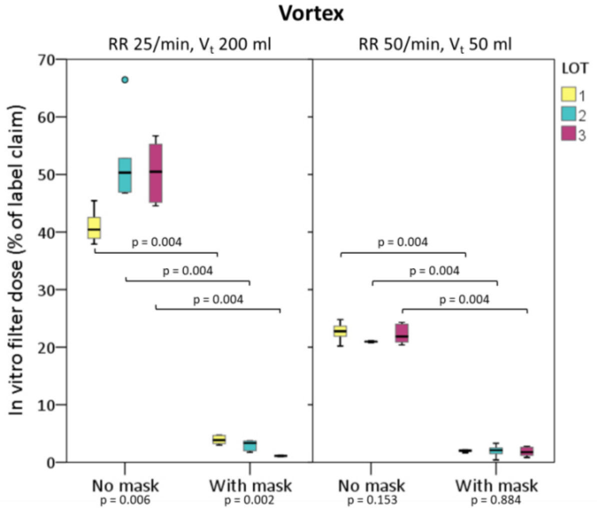

RR, respiratory rate. Vt, tidal volume.

LOT, production batch. P-values shown above the bars refer to differences with or without a mask, based on the Mann–Whitney U test.

P-values below the graph refer to differences between devices from different production batches, based on the Kruskal–Wallis test.

Figure 1. Study timeline in the emergency department

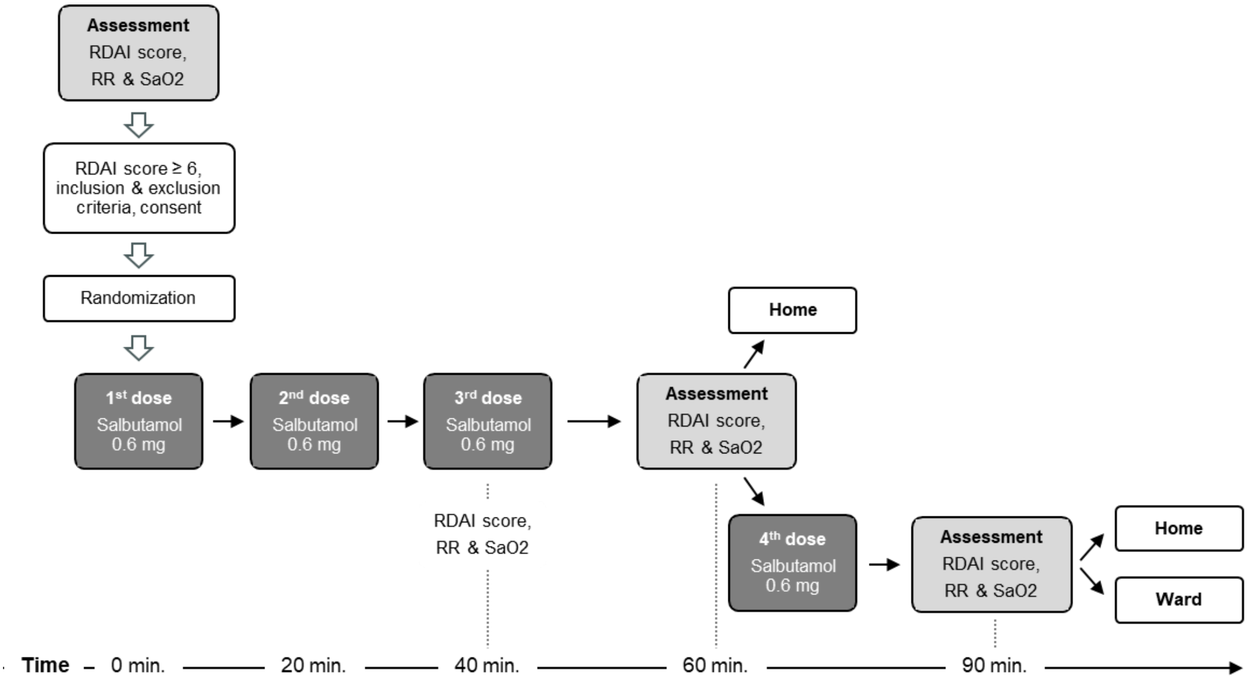

Rationale for Study Design and Methodological Choices in the CHAMBER

Target Population

The aim is to collect a study population as homogeneous as possible in terms of obstructive symptoms. Children under 6 months of age primarily suffer from bronchiolitis, which has been shown to respond poorly to bronchodilator therapy. Bronchiolitis refers to inflammation of the terminal bronchioles in infants. In children over 4 years of age, obstruction is more frequently associated with asthma that typically requires regular medication. Therefore, the study will recruit children aged 6 months to 4 years, focusing primarily on treating symptoms triggered by viral infections in this age group.

Sample Size

Based on previous studies [12],[13] and clinical experience, we estimate that the average RDAI score for children presenting to the emergency department is 11 and that this score decreases to approximately 5 after treatment. The primary outcome is the difference in post-treatment RDAI score between the groups. A difference of at least 2 points is considered clinically meaningful. The sample size is calculated to detect a minimum 2-point difference in final RDAI score (measured at the time of treatment outcome decision) with 90% statistical power (β-error 10%) and 95% confidence (α-error 5%). The between-group difference in RDAI scores will be analyzed using a t-test, and the standard deviation is assumed to be 2.5 based on earlier studies. Thus, 33 children per group are needed for the final analysis. We anticipate that approximately 15% of enrolled children will be excluded from the analysis due to protocol deviations (e.g., adverse effects, insufficient efficacy, or poor treatment adherence). Therefore, 40 children will be recruited per group, for a total of 80 participants.

Statistical analysis

All statistical analyses will be conducted in the intention-to-treat population based on predefined outcome measures, comparing children treated with each of the two valved holding chambers. Continuous variables with normal distribution will be analyzed using the unpaired *t*-test, and non-normally distributed data using the Mann–Whitney *U* test. Differences in proportions will be assessed with the standard normal deviate (*Z*) test for proportions. Results will be presented as proportions, risk differences, risk ratios, and numbers needed to treat (NNT), each with corresponding 95% confidence intervals (CIs). *Amended in 25.2.2021, new analysis added:* A sensitivity analysis will be performed excluding children with poor cooperation, defined as a mean cooperation score below 4.

Ethical Considerations

All participants will receive currently recommended treatment. Half of the children will receive bronchodilator medication using the spacer most commonly used in current clinical practice, while the other half will use an officially approved alternative device that, based on *in vitro* studies, appears to outperform the standard device. Both treatments are expected to be beneficial. The study's purpose and significance are explained both orally and in writing, and the parents of all participating children will sign a written informed consent form. Declining participation will not affect the child's medical care. No additional blood samples or painful procedures are included in the study. Any potential adverse events will also be monitored.

The regional ethics committee of the Tampere University Hospital special responsibility area has approved the study on the 18th of March 2019 (Ethics Committee Code: **R19030**). The study will be registered in the *ClinicalTrials.gov* database prior to the enrolment of the first participant. Experts from Fimea and Valvira have reviewed the study. In the CHAMBER study, salbutamol will be used according to its approved indication for patients who would otherwise receive a bronchodilator, and no procedures are performed solely for the purposes of the study. For this reason, no separate notification to Fimea is required. Since both spacer devices are CE-marked and approved for the intended use and age group, no separate notification is required for Valvira either.

Significance of the Study

Spacer devices are currently used under the assumption that their functional performance is equivalent. However, in vitro study results suggest that devices in use in Finland may differ significantly in terms of drug delivery efficiency.

One of the key goals of the National Allergy Program 2008–2018 was to concentrate resources on preventing severe symptoms and asthma exacerbations [14]. Airway-obstructive symptoms remain a very common issue in children, and reducing their frequency and treating acute episodes effectively is essential for reducing both suffering and healthcare costs. It is particularly important to evaluate, develop, and standardize key treatment practices.

References

1. Csonka P, Mertsola J, Kaila M, Ashorn P. Regional variation in the diagnosis of asthma among preschool-age children. *Pediatr Allergy Immunol* 2000; 11: 189–192.
2. Martinez FD, Wright A, Taussig L, Holberg C, Halonen M, Morgan W. Asthma and wheezing in the first six years of life. *N. Engl. J. Med.* 1995; 332: 133–138.
3. Cates CJ, Welsh E, Rowe B. Holding chambers (spacers) versus nebulisers for beta-agonist treatment of acute asthma (Review). *Cochrane Database Syst. Rev.* [Internet] 2013; 13: CD000052Available from: https://www.cochranelibrary.com/cdsr/doi/10.1002/14651858.CD000052.pub3/full.
4. Mandelberg A, Tsehori S, Houri S, Gilad E, Morag B, Priel IE. Is nebulized aerosol treatment necessary in the pediatric emergency department? Comparison with a metal spacer device for metered-dose inhaler. *Chest* 2000; 117: 1309–1313.
5. Deerojanawong J, Manuyakorn W, Prapphal N, Harnruthakorn C. Randomized Controlled Trial of Salbutamol Aerosol Therapy Via Metered Dose Inhaler-Spacer vs . Jet Nebulizer in Young Children With Wheezing. 2005; 472: 466–472.
6. Delgado A, Chou KJ, Johnson Silver E, Crain EF. Nebulizers vs metered-dose inhalers with spacers for bronchodilator therapy to treat wheezing in children aged 2 to 24 months in a pediatric emergency department. *Arch. Pediatr. Adolesc. Med.* 2003; 157: 76–80.
7. Castro-Rodriguez JA, Rodrigo GJ. β-agonists through metered-dose inhaler with valved holding chamber versus nebulizer for acute exacerbation of wheezing or asthma in children under 5 years of age: A systematic review with meta-analysis. *J. Pediatr.* 2004; 145: 172–177.
8. Suomalaisen Lääkäriseuran Duodecimin, Suomen Keuhkolääkäriyhdistys ry:n SL ry:n ja SKFY ry:n asettama työryhmä. Astma Käypä hoito -suositus [Internet]. Duodecim 2012. p. 1–30Available from: http://www.kaypahoito.fi/web/kh/suositukset/suositus?id=hoi06030.
9. Turpeinen M, Nikander K, Malmberg LP, Pelkonen AS, Turpeinen M, Nikander K, Malmberg LP PA. Metered Dose Inhaler Add-On Devices: Is the Inhaled Mass of Drug Dependent on the Size of the Infant? *J. Aerosol Med.* [Internet] 1999; 12: 171–176Available from: http://www.liebertpub.com/doi/10.1089/jam.1999.12.171.
10. Dissanayake S, Nagel M, Falaschetti E, Suggett J. Are valved holding chambers (VHCs) interchangeable? An in vitro evaluation of VHC equivalence. *Pulm. Pharmacol. Ther.* [Internet] Elsevier Ltd; 2018; 48: 179–184Available from: https://doi.org/10.1016/j.pupt.2017.10.005.
11. Minh KT, von Hollen D, von Königslöw AJ, Nikander K, Janssens HM. An Instrumented Valved Holding Chamber with Facemask to Measure Application Forces and Flow in Young Asthmatic Children. *J. Aerosol Med. Pulm. Drug Deliv.* [Internet] 2014; 27: S-55-S-62Available from: http://online.liebertpub.com/doi/abs/10.1089/jamp.2014.1129.
12. Menon K, Sutcliffe T, Klassen TP. A randomized trial comparing the efficacy of epinephrine with salbutamol in the treatment of acute bronchiolitis. *J. Pediatr.* 1995; 126: 1004–1007.
13. Pruikkonen H, Tapiainen T, Kallio M, Dunder T, Pokka T, Uhari M, Renko M. Intravenous magnesium sulfate for acute wheezing in young children: a randomised double-blind trial. *Eur. Respir. J.* [Internet] 2018; 51: 1701579Available from: http://erj.ersjournals.com/lookup/doi/10.1183/13993003.01579-2017.
14. Haahtela T, Hertzen L Von, Mäkelä M, Hannuksela M. Kansallinen allergiaohjelma 2008–2018 – aika muuttaa suuntaa. 2008; : 9–21.
