# Supplementary material for: Valved Holding Chambers in Young Children With Acute Wheezing: A Randomized Clinical Trial
Source: JAMA Pediatr. 2026 Feb 23;180(5):567–74. doi: 10.1001/jamapediatrics.2025.6479 (PMC12931466; doi:10.1001/jamapediatrics.2025.6479)
Supplement: Supplement 3. — Data sharing statement [file jamapediatr-e256479-s003.pdf]

Data Sharing Statement

Csonka. Valved Holding Chambers in Young Children With Acute Wheezing. *JAMA Pediatr*. Published February 23, 2026. doi:10.1001/jamapediatrics.2025.6479

Data

Additional Information: https://clinicaltrials.gov/study/NCT03900494 ClinicalTrials.gov number NCT03900494
Data available: Yes
Data types: Deidentified participant data
How to access data: Individual de-identified participant data (including data dictionary), statistical code, and related materials are not publicly available due to Finnish legislation, which prohibits open access to patient data, even in de-identified form. However, data supporting the findings of this study may be made available upon reasonable request for clinical research purposes from the corresponding author, subject to applicable ethical and legal approvals.
When available: With publication

Supporting Documents

Document types: Statistical/analytic code, Informed consent form
How to access documents: Individual de-identified participant data (including data dictionary), statistical code, and related materials are not publicly available due to Finnish legislation, which prohibits open access to patient data, even in de-identified form. However, data supporting the findings of this study may be made available upon reasonable request for clinical research purposes from the corresponding author, subject to applicable ethical and legal approvals.
When available: With publication

Additional Information

Who can access the data: Individual de-identified participant data (including data dictionary), statistical code, and related materials are not publicly available due to Finnish legislation, which prohibits open access to patient data, even in de-identified form. However, data supporting the findings of this study may be made available upon reasonable request for clinical research purposes from the corresponding author, subject to applicable ethical and legal approvals.
Types of analyses: All data.
Mechanisms of data availability: By contacting the PI, peter.csonka@tuni.fi
Any additional restrictions: Individual de-identified participant data (including data dictionary), statistical code, and related materials are not publicly available due to Finnish legislation, which prohibits open access to patient data, even in de-identified form. However, data supporting the findings of this study may be made available upon reasonable request for clinical research purposes from the corresponding author, subject to applicable ethical and legal approvals.
